# Supplementary material for: A Repetitive Acipenser gueldenstaedtii Genomic Region Aligning with the Acipenser baerii IGLV Gene Cluster Suggests a Role as a Transcription Termination Element Across Several Sturgeon Species
Source: Int J Mol Sci. 2024 Nov 26;25(23):12685. doi: 10.3390/ijms252312685 (PMC11640988; doi:10.3390/ijms252312685)
Supplement: Supplementary file 1 [file ijms-25-12685-s001.zip › Supplementary Data_Table.docx]

**S Table 1.**

| Primer name | Direction | Sequences |
| --- | --- | --- |
| H7 | N/A | **CTGCCACGAG** |
| M5-1F  M5-1Rn | Forward  Reverse | gct**C**gaggtcatga**T**g**T**tg**gc**  **ggatccctactctagccataatct**gc |
| B-IF-for  B-IF-rev | Forward  Reverse | **gcgcatgatatcTctCttgcgg**  **TGTGGCTATTgcAgCgTgc** |
| A ba-3411R | Reverse | actatgtccattgcaatgcgctc |
| CMV-for | Forward | gtaggcgtgtacggtgggag |
| IGLV3551-3573-1F | Forward | agagtag**gga tcc**actggac aac |
| IGLV8806-8784-2R | Reverse | agagtc **aagctt** tgttg ttgtggattc tgttgcc |
| IGLV5276-5233-3R | Reverse | gagttcagagtgctatccagtacatcatggaaccaaaggtgaag |
| IGLV5233-5276-4F | Forward | cttcacct ttggttccat gatgtactgg atagcactct gaactc |
| IGLV7018-6975-5R | Reverse | gaattgaaatgaatgtgaagctatacttcaggaagatttctcag |
| IGLV6975-7018-6F | Forward | ctgaga aatcttcctg aagtatagct tcacattcat ttcaattc |
| M1-fora | Forward | ttagtgaggtggcgcgatatg |
| M1-rev | Reverse | acttttgaccggtagtgtatataattc |
| M1-reva | Reverse | gtactctgaaattaaagcatagaactt |
