# Supplementary material for: A Repetitive Acipenser gueldenstaedtii Genomic Region Aligning with the Acipenser baerii IGLV Gene Cluster Suggests a Role as a Transcription Termination Element Across Several Sturgeon Species
Source: Int J Mol Sci. 2024 Nov 26;25(23):12685. doi: 10.3390/ijms252312685 (PMC11640988; doi:10.3390/ijms252312685)
Supplement: Supplementary file 1 [file ijms-25-12685-s001.zip › Supplementary Figure and Table Legends.docx]

**Figures and Table legend**

**S Figure S1.** PCR amplification using AllWSex2/Ag49 primer sets and DNA from the adult Russian sturgeon. Lanes 1-5: adult females DNA (F1 through F5); Lanes 6-10: adult males DNA (M1 through M5); Lanes 11-13: DNA from the individual caviar grains originated at the MF. L: 2-log DNA ladder (NEB).

**S Figure S2.** PCR amplification using primers based on the VAC-1M sequence and DNA from the individual caviar grains vs. skin swab samples. Lanes 1-4, MF caviar DNA; lanes 5-14, MF skin swabs DNA that originated from fish of different ages. L: 2-log DNA ladder (NEB).

**S Figure S3.** PCR amplification using AllWSex2/Ag49 primer sets and DNA from the individual caviar grains vs. skin swab samples. Lanes 1-4, MF caviar DNA; lanes 5-14, the same as shown in S Figure 2 skin swabs DNA that originated from the MF fish of different ages. L: 2-log DNA ladder (NEB).

**S Figure S4.** PCR amplification using B-IF-for/B-IF-rev primers and DNA isolated from the skin swab samples, MF. Lanes 1-4 represent MF fish #26, 27, 28, and 30; amplification buffer **E**. Lanes 5-8 represent MF fish #26, 27, 28, and 30; amplification buffer **B**. The smallest size bands represent 168/139 bp variations. L: 2-log DNA ladder (NEB).

**S Table S1.** Primers used for amplification/sequencing. The color coding for some of the primers as well as the type of letters used (capital vs small vs bold) is the same as shown in the corresponding figures.
