# Supplementary material for: A Repetitive Acipenser gueldenstaedtii Genomic Region Aligning with the Acipenser baerii IGLV Gene Cluster Suggests a Role as a Transcription Termination Element Across Several Sturgeon Species
Source: Int J Mol Sci. 2024 Nov 26;25(23):12685. doi: 10.3390/ijms252312685 (PMC11640988; doi:10.3390/ijms252312685)
Supplement: Supplementary file 1 [file ijms-25-12685-s001.zip › Supplementary Materials_MF_DNA_OD.docx]

**Supplementary Materials**

Recently, an international group of scientists have discovered the female-specific region in sturgeon DNA genome resulting in similar and specific for 6 different sturgeon species (*A. sturio*, *A. oxyrinchus*, *H. huso*, *A. ruthenus*, *A. baerii*, and *A. gueldenstaedtii*) PCR product of about 100 bp generated after using the AllWSex2 primer set [5]. Although this region of the sturgeon genome was not associated with any of the specific genes known to regulate fish sex differentiation, the discovery finally opened real practical possibility to design a DNA-based sex diagnostic test for these sturgeon species that can be applied during the early stages of their development. Independently and in parallel from the above-mentioned breakthrough in sturgeon genetics research, we have decided to explore the possibility of discovering any specific for the Russian sturgeon DNA differences using random PCR amplification.

Fresh tissue samples were collected at the Marshallberg farm, Smyrna NC, from fish with known sex characteristics (5 females and 5 males) and submitted to the Louisiana State University GeneLab Core Sequencing Facility (fee for the service) for DNA extraction, PCR-based analysis, cloning, and conventional DNA sequencing. The gel picture of the PCR products generated after using one of the random 10 bases long oligo (H7: CTGCCACGAG) and all 5F and 5M-derived DNA samples is shown in Figure 1. All male DNA samples generated a distinct band on the agarose gel migrating slightly above the corresponding 700 bp long band of the ladder. Interestingly, M1 DNA sample produced a wider band (later found to be a mix band of different sizes). More importantly, the corresponding bands for F1, F3, and F5 DNA samples were completely missing while F2 and F4 generated what appeared to be slightly smaller band size (later found to be 39 bp shorter than the corresponding male PCR products.

F2 and F4 PCR products together with M2, M3, M4, and M5 bands described above were cut from the gel, purified, cloned and used for conventional DNA sequencing. Four individual clones derived from each band were used for capillary DNA sequencing. All 8 female-derived sequences (4 for F2 and 4 for F4) were identical and 696 (676 without counting H7 sequence on both sides of the PCR product) bp long in comparison to the 735 (715 w/o H7 sequence) bp long male-derived sequences designated as VAC-1M (14 clones out of 16 clones sequenced). Two other cloned DNA fragments that derived from M5, were similar in size but totally different from VAC-1M sequence and were designated as VAC-2M. Specific forward primer M1-fora (nts 160-180 with regard to 735 bp VAC-1M sequence) and two specific reverse primers: M1-rev (nts 524-498, located within the respective 39 bp deletion), and M1-reva (nts 610-584, located just outside the deletion) were designed. The same DNA samples as for the random PCR were used and the results are shown in Figure 2. As expected, a single band of 411 bp long was detected for the samples derived from F2 and F4 DNA and no PCR products were detected when the entire corresponding region was missing (F1, F3, and F5). Also, as expected, M2, M3, M4, and M5 samples generated PCR products of 364 bp and 450 bp long based on the different locations of rev and reva primers. Interestingly, M1 sample produced all 3 bands (the middle band corresponding to the female genotype with 39 bp deletion within the VAC-1M sequence) and thus confirming the presence of both genetic variants within the same fish**.**

Considering the significance of the brand-new information about potential use of the AllWSex2 primer set for the early sturgeon sex diagnostics, we decided to test these recently published primers sequences against the same *A. gueldenstaedtii* DNA samples originated from the adult fish that were available. The only problem of using AllWSex2 primer set as published under field conditions would be the real possibility of dealing with false negative PCR results when samples with no PCR product outcome would be automatically assigned as males. Under the field conditions, no product outcome can easily result from either low DNA quality, or no DNA isolated at all (very important factor for the field-based test). We have modified the use of AllWSex2 primers to include Russian sturgeon specific primers to always have an internal DNA template quality control. Ag49 tetrasomic microsatellite loci-based primers set [21], specific for the Russian sturgeon, and producing allele size range between 198 to 219 bp on the gel was used to cover the void. Figure S1 represents the use of AllWSex2 primers in combination with Ag49 primer set. Similarly to Figures S1 and S2, numbers 1 through 5 represent same adult female DNA samples, 6 through 10 represent same adult male DNA samples, and 11 through 13 represent DNA samples isolated from the individual caviar grains derived from randomly selected fish grown at the Marshallberg farm. All adult female-derived DNA samples generated two bands of expected size, while male-derived samples generated a single band corresponding to the Russian sturgeon-specific PCR product. Unfertilized caviar DNA samples produced both female-specific and sturgeon-specific PCR products (Figure S1, lanes 11-13). Initial validation and side-by-side comparison AllWSex2/Ag49 vs VAC-1M-based primers were performed using adult fish; however, the goal is to apply this genetic test to fish of different ages, including juveniles. PCR using VAC-1M -specific primers and DNA from individual caviar grains (Figure S2, lanes 1-4) produced similar bands on the gel with the sizes that would be expected from the corresponding unchanged 735 bp long male VAC-1M sequence. Furthermore, most of the skin swab DNA samples originated from fish of different ages did not show any difference, while lane 9 sample did not generate any PCR product. The same DNA using AllWsex2/Ag49 primers identifies this sample as derived from the male (Figure S3, line 9). All potential female samples generated two bands of predicted size while all male samples produced only one band, respectively (Figure S3, lanes 5-14; multiplex of AllWSex2 and Ag49 primer sets). Importantly, detection of the female-specific band after using DNA samples derived from the individual unfertilized caviar grains (lanes 1-4, Figure S3) strongly points toward female-specific (WZ/ZZ) type of sex determination in sturgeons. We also tested DNA isolated from the commercially available individual caviar grains that were originated from the fish grown at a fish farm in Israel and Evans Fish Farm, FL with very similar outcome (data not shown). DNA samples originated from the skin swabs (lanes 5-14 on both Figures S2 and S3) were isolated from the fish that produced inconclusive sex results by ultrasound testing at the Marshallberg Farm. However, genetic testing using AllWsex2/Ag49 primers was able to pick up 4 potential females (lanes 6, 10, 11, and 12) that would be, most likely, eliminated from consideration under normal conditions. Therefore, these data do not support the use of VAC-1M sequence as a sex-specific genetic marker and all further genetic testing with regard of the juvenile fish will be conducted using AllWsex2/Ag49 multiplex primers set.
