# Supplementary figures and images for: A Repetitive Acipenser gueldenstaedtii Genomic Region Aligning with the Acipenser baerii IGLV Gene Cluster Suggests a Role as a Transcription Termination Element Across Several Sturgeon Species
Source: Int J Mol Sci. 2024 Nov 26;25(23):12685. doi: 10.3390/ijms252312685 (PMC11640988; doi:10.3390/ijms252312685)

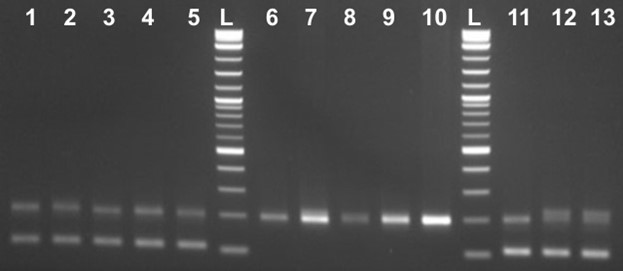

Supplement: Supplementary file 1 [file ijms-25-12685-s001.zip › S1.tiff]

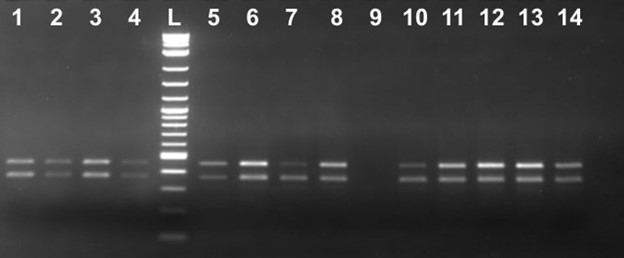

Supplement: Supplementary file 1 [file ijms-25-12685-s001.zip › S2.tiff]

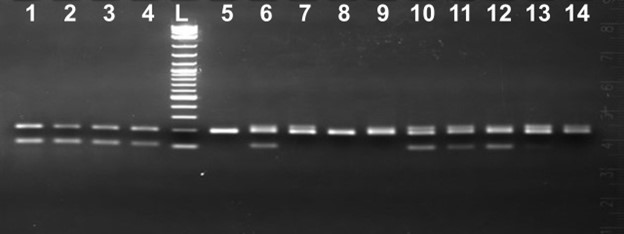

Supplement: Supplementary file 1 [file ijms-25-12685-s001.zip › S3.tiff]

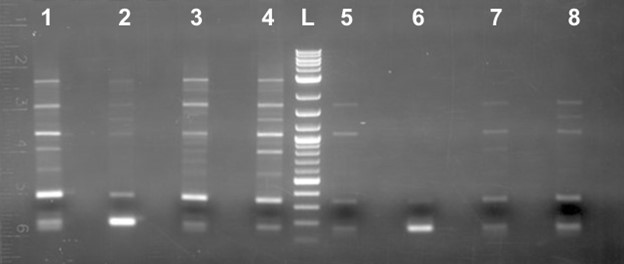

Supplement: Supplementary file 1 [file ijms-25-12685-s001.zip › S4.tiff]
